# Learning strategies scale: adaptation to Portuguese and factor structure

**DOI:** 10.1186/s41155-018-0092-1

**Published:** 2018-06-07

**Authors:** Lara Barros Martins, Thaís Zerbini, Francisco J. Medina

**Affiliations:** 1 Health School (Psychology), IMED – Faculdade Meridional, R. Senador Pinheiro, 304 - Vila Rodrigues, Passo Fundo, RS CEP 99070-220 Brazil; 2 Psychology Department, Laboratório de Psicologia Organizacional e do Trabalho – LabPOT, FFCLRP/Universidade de São Paulo, University of Sao Paulo, Av. Bandeirantes, 3900, Ribeirão Preto, SP CEP 14040-901 Brazil; 3 Social Psychology Department, University of Seville, s/n, Calle Camilo José Cela, 41018 Seville, Spain

**Keywords:** E-learning, Assessment, Learning strategies, Scale, Training transfer, Job performance

## Abstract

Since learning strategies seem to be an important set of variables to explain the effectiveness of training and e-learning in organizations is here to stay, this paper aimed to analyze the factor structure and psychometric properties of a Learning Strategies Scale (LSS) and its relationship with the training transfer in an e-learning corporate context. A total of 3600 employees of a Brazilian bank participated in this study by responding to the LSS after taking part in an online course. We measured training transfer with self-evaluation and hetero-evaluation scales. Internal consistency, confirmatory factor analysis, and multiple regressions were conducted. A four-factor structure and an acceptable level of fit for the model were found. All types of learning strategies were related to training transfer in self-evaluation, and the cognitive and help-seeking strategies contributed to explain training transfer in hetero-evaluation. As a reliable and valid measure that predicts the effectiveness of training and job performance, participants should be advised about the learning strategies that produce better performance results at the workplace. Future research should use it in different contexts and samples, analyzing its relationships with other workplace variables.

## Background

E-learning programs, which consist of using electronic resources to deliver contents through the Internet, along with the rapid technological changes—above all Information and Communication Technologies (ICT)—demand a range of different skills, tending to offer trainees increasingly larger amounts of control over their own learning process (DeRouin, Fritzsche, & Salas, 2005). Hence, “learning how to learn” is strategic nowadays and providing trainees with skills that help them learn and achieve better performance results is especially relevant when contents are available anywhere and anytime (Badia & Monereo, 2010; Warr & Allan, 1998).

In this sense, trainees use procedures called learning strategies to facilitate acquisition, retention, and subsequent application of the knowledge learned in educational programs. These strategies include a set of complex cognitive, behavioral, and self-regulatory skills, which are adapted to the context, consciously and intentionally applied, in order to achieve specific learning goals (Badia & Monereo, 2010; Beluce & Oliveira, 2012). Therefore, those who have been trained in the most effective strategies can achieve better performance results (Tannenbaum & Yukl, 1992; Wexley, 1984), not only in school settings, but throughout life (Bjork, Dunlosky, & Kornell, 2013).

As today’s virtual learning scenarios require continuous management of information and its transformation into knowledge (Badia & Monereo, 2010), understanding whether (and how) learning strategies are related to knowledge acquisition and performance is critical (Holman, Epitropaki, & Fernie, 2001). In addition, growing empirical evidence suggests the importance of self-regulatory strategies in e-learning (Vovides, Sanchez-Alonso, Mitropoulou, & Nickmans, 2007), their mediation role in the relationship between training and learning (Aguinis & Kraiger, 2009), and their relevance to explain training transfer, providing trainees with skills that help them transfer successfully when they return to the workplace (Burke & Hutchins, 2007).

Warr and Allan (1998) proposed a classification of learning strategies that divides them into three components: (1) cognitive (rehearsal, organization, elaboration), (2) behavioral (interpersonal and written help-seeking, practical application), and (3) self-regulatory (emotion and motivation control, comprehension monitoring).

Cognitive learning strategies comprise mental repetition of original information; creation of mental schemes that relate elements to be learned; and connections between course material-previous knowledge and its implications. Behavioral learning strategies consist of proactive behavior to search for help among peers or instructors, or from any source that does not involve social contact, besides the practical application of newly learned skills or behaviors. Self-regulatory strategies include emotion control, motivation control, and monitoring of comprehension processes, which express the learner’s control of the anxiety, concentration, attention, motivation, and the learning process itself.

Based on this taxonomy, studies have been conducted and empirical evidence indicates the main importance of cognitive and behavioral strategies for learning and transferring in organizational contexts, including when trainees were enrolled in e-learning courses (Brandão & Borges-Andrade, 2011; Crouse, Doyle, & Young, 2011; Pantoja & Borges-Andrade, 2009; Warr & Downing, 2000; Zerbini & Abbad, 2010a). Research points that the learning strategies used by adults in the workplace are similar to those used in previous educational settings (Holman, Epitropaki, & Fernie, 2001), maybe due to the fact that individuals, as learners, acquire a repertoire of learning strategies throughout their academic lives, reproducing them in the adulthood and at work, even if they participate in online courses.

In technical courses, cognitive strategies (mental repetition and active reflection), behavioral (written help-seeking and practical application), and self-regulatory (emotion and motivation control) were positively related to changes in knowledge (Warr & Downing, 2000). The mental repetition and behavioral strategies were positively related to training transfer (Warr & Allan, 1998), so were elaboration and comprehension monitoring (Zerbini & Abbad, 2008, 2010b). Interpersonal help-seeking was related to the acquisition, retention, and transfer of new skills (Brandão & Borges-Andrade, 2011; Pantoja & Borges-Andrade, 2009), along with the practical application strategies (Crouse, Doyle, & Young, 2011; Pantoja, 2004; Zerbini & Abbad, 2005).

According to Salas, Tannenbaum, Kraiger, and Smith-Jentsch (2012), the practical opportunities to perform during training must enable trainees to engage in cognitive processes similar to those demanded when they return to work. Perhaps that is a reason why cognitive and behavioral strategies play an important part when considering corporate environments, independently on the training design, because the pattern of use of learning strategies seems to depend more on the skill to be developed and the task to be performed—which in work activities are mainly cognitive and behavioral. Still, trainees that are also workers search mostly the training content utility and applicability, and such strategies enable them to analyze the material, seek for connections with their previous knowledge and the implications to their daily situations at work.

Similarly, self-regulatory strategies have shown positive and strong relationships with good results and academic success in e-learning environments (Aguinis & Kraiger, 2009; Johnson, Gueutal, & Falbe, 2009; Martins & Zerbini, 2016; Vovides, Sanchez-Alonso, Mitropoulou, & Nickmans, 2007; Warr & Bunce, 1995; Warr & Downing, 2000). The use of self-regulatory processes seems to be quite adequate in an online context, while studying in the workplace may require more effort, focused attention, and self-monitoring of learning. Comparing to face-to-face training, trainees, who study at a distance, more frequently need to force themselves to pay attention and keep their interest and concentration in the learning lesson. Moreover, it requires skills that make it possible to combine work activities with studying and performance goals with learning needs.

The differences among empirical results indicate that the particular context and the specificities of the sample might demand the use of certain learning strategies rather than others. Consequently, there would not be a single best strategy, but more effective alternatives that may vary according to different learning contexts (Levin, 1986). Therefore, the types of learning strategies that are more effective to explain the training outcomes might vary depending on the organization, the training design, and the sample.

First, different business segments may influence the adoption of certain learning strategies. Second, the nature of the course (related to cognitive, affective, or psychomotor skills; theoretical or practical), the complexity of its learning goals, and the delivery mode (e-learning, blended, or face-to-face) will possibly determine which are the best learning strategies to draw on during the training process. Third, the individual characteristics of the sample (age, gender, level of education, etc.) and occupational specificities (nature and complexity of the job, types of activities, and attributions of work) may also affect this choice (Brandão & Borges-Andrade, 2011).

Although learning strategies seem to be an important set of variables to explain the effectiveness of training (Zerbini & Abbad, 2010a), there are few studies centered on this matter (Burke & Hutchins, 2007), especially in e-learning settings and in corporate courses. Moreover, once most of research focus on traditional educational contexts, outcomes have been limited to learning or only indirectly tested relationships to transfer (Burke & Hutchins, 2007). An important line of research on training and learning strategies exists in Brazil (Borges-Ferreira, 2005; Brandão & Borges-Andrade, 2011; Martins & Zerbini, 2014; Pantoja & Borges-Andrade, 2009; Zerbini & Abbad, 2008); nevertheless, the translated instruments are not validated, since mainly exploratory research was conducted; besides, there are no valid and reliable scales for online training.

Thus, this study aims to validate a Learning Strategies Scale (LSS) in Portuguese, by analyzing its factor structure and psychometric properties. In addition, its concurrent validity was evaluated by testing the relationship between the LSS and training transfer in an e-learning corporate context. Training transfer refers to the effective application at the workplace of the new competencies acquired during a training program (Bell, Tannenbaum, Ford, Noe, & Kraiger, 2017; Grossman & Salas, 2011). It has been the main performance indicator of the effectiveness of training at the individual level, as proposed by important models of training evaluation in the literature (Baldwin & Ford, 1998; Borges-Andrade, 2006; Hamblin, 1978; Kirkpatrick, 1976). The LSS makes it possible to investigate the learning strategies in several contexts, both in organizations and higher education institutions.

## Methods

### Participants

The participants were employees of a large public Brazilian bank that have taken, at the workplace, the online “Operational Efficiency” training, with the objective of identifying ways to promote operational efficiency in work activities at the company. Besides, managers have evaluated the influences of that course on their subordinates’ work behaviors. The answers obtained as to the demographic characterization, from 1639 employees and 2261 managers, respectively, show that most of the sample were men (56.8 and 67.7%), ranging in age from 46 to 55 years old (26.1 and 41.7%), which have been in their present post for 1 to 3 years (20.3 and 27.5%), in the area that supports business and management activities at the bank (37.8 and 59.1%), and who held a graduate degree or more (63.3 and 86.3%).

### Instruments

The Learning Strategies Scale (LSS) is a 20-item self-report questionnaire, using a 5-point Likert scale—response alternatives scored from 1 to 5, meaning respectively, “never” and “always”—to know how frequently the participants used the procedures to help them to learn during a specific learning program or training. It was adapted from the eight-factor and 45-item original version in English (Warr & Downing, 2000) that was first built to suit academic contexts and was tested in a sample of adults who had taken technical courses.

Previously in Brazilian studies, based on Warr and Downing’s scale, a 28-item version (seven-factor structure, .75 < *α* < .89) without the self-regulatory strategies was used by Zerbini and Abbad (2008) to evaluate an open distance course; then, it was adapted again (29-item version, four-factor structure, .68 < *α* < .90) to fit a higher education context (Martins & Zerbini, 2014). The maintenance of the good psychometric properties of these scales supports the decision of excluding items from the original scale, keeping their validity evidences.

Nevertheless, some reasons that motivated the proposition of a new adapted version refer mainly to the following: (i) the measures still contained some items similar in content that measured the same aspects (e.g., “I pushed myself even harder when I began to lose interest” and “I increased my effort when I began to lose interest”); (ii) some items were more appropriate for traditional designs (face-to-face instructions) or expressed procedures not often used in e-learning—for example, thinking out questions or setting oneself tests to check the understanding of content or giving too much relevance to written materials; (iii) were applied only in academic contexts, not focusing on online trainings, neither taken by workers nor in organizations; and (iv) studies did not perform confirmatory factor analysis on their empirical structures.

Furthermore, considering the characteristics of an online instructional design and the specific context of application, a more parsimonious scale was needed. Therefore, a synthetic version is proposed: we made some changes in the instrument, reducing its number of items and points of the rating scale (from 11 to 5 points), so it would fit better e-learning and be more appropriate to corporate contexts. Once the evaluation of training programs are usually conducted, by researchers or practitioners, at the workplace during the working hours, instruments must be easy and fast to respond. Items have been grouped according to the four-factor structure identified in this study, but they should be intermingled in use (see Table 1 in the annexes).

**Table 1:** Learning Strategies Scale

Item	Portuguese	English*
Cognitive and help-seeking		
7	Busquei auxílio de colegas para esclarecer minhas dúvidas sobre os conteúdos do curso.	I asked other course members for help when I did not fully understand the material.
8	Busquei solucionar minhas dúvidas ao consultar os materiais didáticos do curso.	I filled in gaps in my knowledge by getting hold of some written material.
9	Busquei compreender melhor os conteúdos ao estudá-los nos materiais didáticos do curso.	I tried to understand something better by locating and studying a relevant document.
10	Busquei outras fontes de pesquisa relacionadas ao curso para me ajudar a aprender.	I sought out relevant documents to help me learn.
13	Li o conteúdo do curso várias vezes como método para aprender.	I read through material several times as a method of learning it.
14	Repeti mentalmente os conteúdos do curso que gostaria de aprender até perceber que havia entendido.	I repeated in my mind things I wanted to learn.
15	Fiz anotações, resumos e/ou esquemas dos conteúdos do curso como método para aprender.	I copied out material in order to help me learn it.
20	Revisei os conteúdos relativos aos exercícios em que cometi erros.	I revised the material about the exercises I made mistakes.
Emotion control		
1	Mantive a calma quando tive dificuldades durante o curso.	I told myself not to worry when things were difficult.
2	Mantive a calma com a possibilidade de ter um rendimento abaixo do esperado.	I tried not to worry about the possibility of doing worse than I wanted.
3	Mantive a calma diante dos erros que cometi ao realizar atividades do curso.	I tried to persuade myself not to worry about mistakes I made.
Elaboration and practical application		
11	Tentei entender o conteúdo ao aplicá-lo na prática, ao invés de dedicar tempo lendo ou pedindo ajuda a alguém.	I learned something by doing it, rather than by studying a book or talking with someone.
12	Realizei os exercícios práticos propostos ao longo do curso para me ajudar a aprender.	I carried out practical exercises to help myself learn.
16	Refleti sobre as implicações que os conteúdos aprendidos poderiam ter.	I thought around new material and its implications.
17	Identifiquei situações diárias em que eu pudesse aplicar os conteúdos do curso.	I identified daily situations where I could try the material out in practice.
18	Busquei desenvolver uma ideia global sobre como os conteúdos do curso se relacionavam entre si.	I tried to develop an overall idea of how different bits of the material relate to each other.
19	Associei os conteúdos do curso aos meus conhecimentos anteriores.	I looked for connections between course material and what I already knew.
Motivation control		
4	Esforcei-me mais quando percebi que estava perdendo a concentração.	When I was feeling bored, I forced myself to pay attention.
5	Esforcei-me mais quando percebi que estava perdendo o interesse no assunto.	I increased my effort when I began to lose interest.
6	Esforcei-me para verificar minha compreensão sobre o que estava sendo ensinado.	I pushed myself even harder to concentrate in the learning lesson.

Item 11, a practical application strategy, did not group with any factor, not remaining in the empirical structure. Item 8, a help-seeking strategy, was eliminated from the factor structure (CFA)

*Items from the original scale (Warr & Downing, 2000)

To measure the effectiveness of training at work, two instruments were used, adapted from the “Training Transfer Scale” (Pilati & Abbad, 2005), one for the self-assessment (*α* = .89; factor loadings .62 to .86) and another for the hetero-assessment (*α* = .94; factor loadings .78 to .90). Both with seven items that measure the training transfer, that is, the indirect influence of training on the overall performance, attitudes, and motivation of trainees; in other words, does training have an impact on the effectiveness of behavior (job performance)? Sample items included the following: “I/The employee can do my/his/her work faster”, “It improved the quality of my/his/her work”, and “It increased my/his/her motivation to work”, with response alternatives scored from 1 “do not agree” to 5 “totally agree”.

### Procedures

Direct translation and adaptation of the items from the original questionnaire were conducted by a Portuguese native speaker researcher that has proficiency in the English language. Then, the accuracy of the translated version was judged by a bilingual professional in a process of reverse translation. The Portuguese version has been revised in a process of semantic analysis, in which graduates (*n* = 4) and later experts in the field (*n* = 4) judged the content validity of the scale. These procedures assure that the content validity of the adapted instrument was not affected by the reduction of items, which was then also corroborated by the statistical validation.

An online application of the instruments to a potential sample of 3600 employees that had participated in the training “Operational Efficiency” was performed. Besides, managers have evaluated the influences of the training on their subordinates’ work behaviors, after approximately 6 months from the end of the training, so its effects could be observed at the individual level. The bank commonly performs this type of evaluation, including both workers and supervisors’ reports, according to this timeframe (6 months after employees had completed the training), so the procedures of this research took place within the regular training evaluation performed by the bank. The self- and hetero-assessment obtained, respectively, an overall return rate of 61.1% (*N* = 2201) and 66.9% (*N* = 2411).

### Data analysis

To run the analyses, the SPSS (Statistical Package for the Social Science) and SPSS AMOS 23.0 were used. Preliminary analyses were done to verify the existence of lost values, univariate and multivariate outliers.

In the confirmatory factor analysis (CFA), normality was assessed by the skewness and kurtosis values of the items, which should be ranged from − 2.0 to 2.0, although the larger the sample size, the less concern about normality should exist. The estimation method used was maximum likelihood, which is very reliable in cases where the distributions of the variables are normal. To judge the model fit, the following goodness-of-fit indices were considered acceptable: when the *χ*^2^/*df* is less than 5, the Goodness of Fit Index (GFI) and the incremental indexes (CFI and TLI) are higher than .90 (ideally above .95), and the error rates (RMSEA and RMSR) are less than .08 (ideally below .05). The smaller the BIC value, the better, as it is a parsimony indicator that compares model fits (Byrne, 2010).

To analyze the concurrent validity of the scale, we employed multiple regression analysis, using the four factors of the LSS as predictors of training transfer.

## Results

### Descriptive analysis

Descriptive statistics are presented in Table 2.

**Table 2:** Means and standard deviations for the Learning Strategies Scale

Item	Mean	Standard deviation
1	4.55	0.63
6	4.42	0.71
4	4.39	0.76
12	4.36	0.83
19	4.29	0.75
3	4.29	0.76
5	4.21	0.86
2	4.17	0.91
17	4.15	0.81
16	4.13	0.84
9	4.13	0.94
18	4.08	0.85
8	4.02	1.04
20	3.88	1.04
11	3.83	1.02
14	3.44	1.13
13	3.35	1.12
15	3.22	1.32
7	2.93	1.35
10	2.82	1.31

Response alternatives 1–2 (never–rarely), 3 (sometimes), 4–5 (often, always)

### Confirmatory factor analysis and internal consistency

With respect to distributional assumptions, skewness (range from − 1.60 to .08) and kurtosis (range from − 1.00 to 1.94) significantly departed from the values expected under the normality assumption for 15 out of 19 items—the exceptions were for items: 1 (3.94), 12 (2.56), 4 (2.74), and 6 (2.43).

The original model fit statistics indicated that some re-specifications would increase its fit (CMIN/DF = 16.0; GFI = .88; RMSR = .06; CFI = .87; TLI = .85; RMSEA = .08). So, this model (four-factor structure, 19 items, and .80 < *α* < .85), with four correlated dimensions, indicated two measurement errors with the highest values, in the pair of items 8 (“I filled in gaps in my knowledge by getting hold of some written material”) and 9 (“I tried to understand something better by locating and studying a relevant document”). Since their contents are very similar, respondents probably understood them as the same measurement. The decision to remove one of them can be theoretically justified, because both refer to the search for understanding content by obtaining the course material. The behaviors related to resolving questions and understanding the contents better are similar: the first (addressing questions) may be contained and expressed in the second (better understanding). Therefore, item 8 was chosen to be eliminated, as it had lower factor loading compared to item 9, there were other error residuals associated with it, and in addition, the overall alpha scale would remain the same with its withdrawal (*α* = .89).

**Fig. 1:**
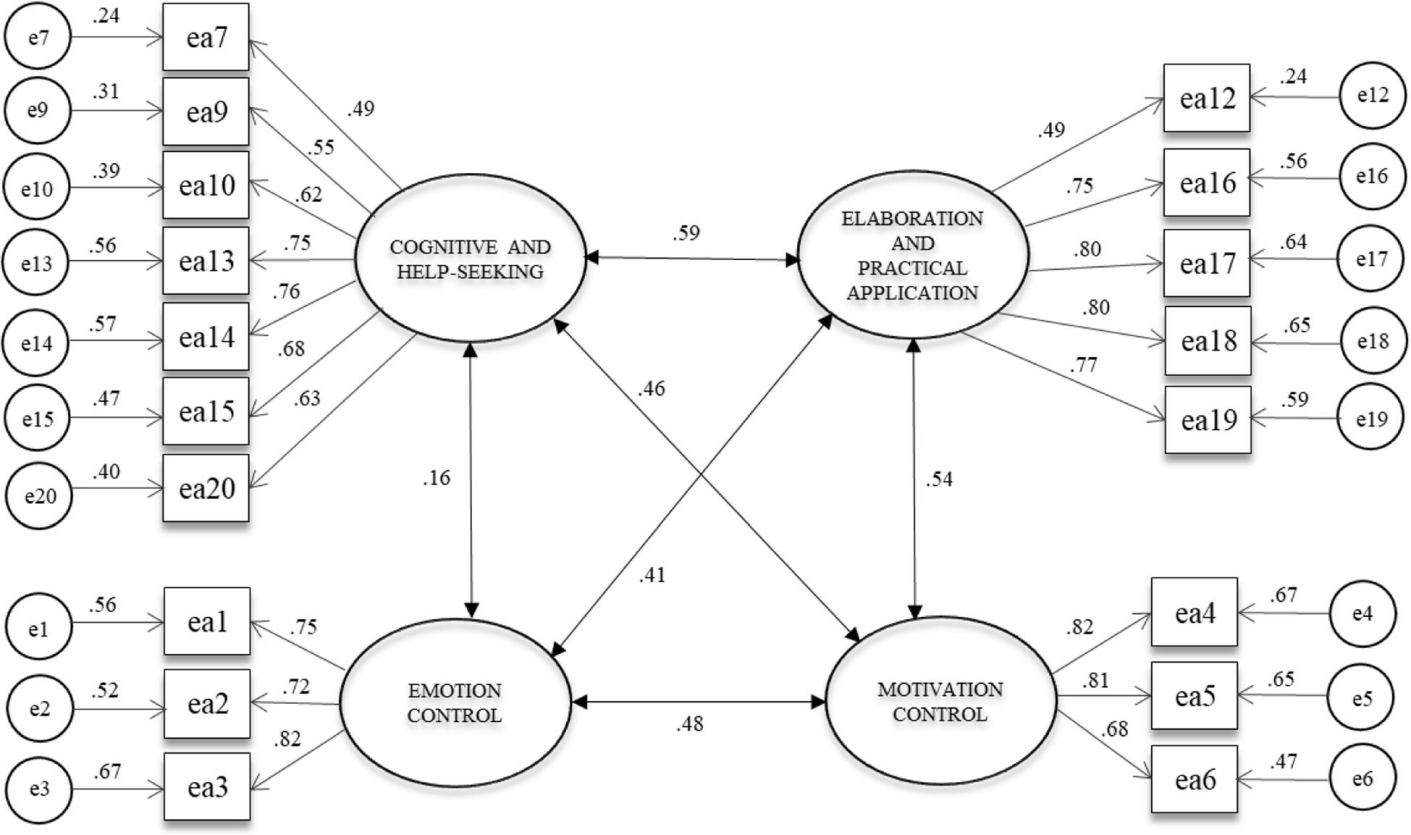
Re-specified model. Standardized factor loadings, correlation coefficients, and standard errors of the confirmatory factor analysis for the Learning Strategies Scale

Fit indices for this re-specified model (Fig. 1) are presented in Table 3, which indicate an acceptable model fit.

**Table 3:** Goodness-of-fit indices for the original and re-specified model

Model	*χ* ^2^	*df*	CMIN/DF	GFI	RMSR	CFI	TLI	RMSEA
Original model	2336.949	146	16.0	.88	.06	.87	.85	.08
Re-specified model	1500.119	129	11.62	.93	.05	.91	.89	.07

*N* = 2071; the re-specified model does not include item 8

The scale has an excellent overall Cronbach’s alpha (*α* = .89), with a total of 18 items that measure the frequency of use of learning strategies—item 8, a help-seeking strategy, was eliminated from the factor structure; and item 11, a practical application strategy, did not group with any factor, not remaining in the empirical structure. Cronbach’s alpha for each dimension and correlations between the four factors of the LSS are presented in Table 4.

**Table 4:** Cronbach’s alpha and correlations between Learning Strategies Scale factors

Variables	*α*	EA1	EA2	EA3	EA4
Cognitive/help-seeking [EA1]	.85	–			
Emotion control [EA2]	.80	.15*	–		
Elaboration/practical application [EA3]	.84	.52*	.38*	–	
Motivation control [EA4]	.81	.45*	.40*	.51*	–
Mean		3.49	4.33	4.20	4.33
Standard deviation		.80	.65	.64	.66

All correlations are significant at *p < .01

### Influence of the learning strategies scale on training effectiveness

Considering the self-assessment of the training, all learning strategies have proved to be important for the explanation of training transfer (*R*^2^ = .28; *p* < .01), mainly the elaboration and practical application strategies (EA3: *β* = .32, *p* < .01). On the other hand, for the hetero-assessment of the training, learning strategies had a less decisive role, contributing only 2% of the variability of training transfer (*R*^*2*^ = .02; *p* < .01). In any case, this explanation is significant and highlights the cognitive and help-seeking—cognitive strategies of rehearsal and organization of learning content and searching for interpersonal help and course material—to help transfer (EA1: *β* = .11, *p* < .01). In a less expressive way, the elaboration and practical application strategies (EA3)—the main predictors of training transfer in the self-assessment—appear as marginally significant in explaining the training transfer in the hetero-assessment. All types of learning strategies are correlated to training transfer (.25 < *r* < .49), particularly the elaboration and practical application strategies (see Table 5).

**Table 5:** Learning strategies as predictors of training transfer

Variables	Self-evaluation	Hetero-evaluation	Correlation with training transfer
Cognitive/help-seeking [EA1]	*β* = .15*	*β* = .11*	*r* = .39**
Emotion control [EA2]	*β* = .05**	–	*r* = .25**
Elaboration/practical application [EA3]	*β* = .32*	*β* = .06^+^	*r* = .49**
Motivation control [EA4]	*β* = .13*	–	*r* = .39**
*R* ^*2*^	*R*^*2*^ = .28*	*R*^*2*^ = .02*	

*β* standardized regression coefficients, *r* correlation coefficients

*p < .01; **p < .05; ^+^*p* < .10

## Discussion

This study has achieved its principal aim providing an original and not previously published scale, with good psychometric characteristics, of easy and simple application. It is a reliable instrument, capable of measuring the frequency of use of learning strategies in several learning contexts, as in higher education and organizations, and is an important diagnosis tool to assess the effectiveness of training at the workplace.

The four-factor solution presents theoretical sense, having been found in a previous study (Martins & Zerbini, 2014), but with a different grouping of some learning strategies. The four factors are the learning strategies of cognitive and help-seeking, emotion control, elaboration-practical application, and motivation control. Other studies have also found structures with a reduced number of factors (Borges-Ferreira, 2005; Brandão & Borges-Andrade, 2011) compared to the original version, in which cognitive strategies were grouped together in the same factor, or even within behavioral strategies.

Only a few studies included the self-regulatory items in the scale, because of the particularities (nature and complexity) of the courses evaluated, or supposing the low influence of this kind of strategy on the explanation of work behaviors—which may be further investigated in future research, due to recent recommendations on this topic (see Aguinis & Kraiger, 2009; Burke & Hutchins, 2007). Results show that maintaining two separate components (emotion and motivation control) of self-regulatory strategies on the factor structure of the present scale—validated in an organizational context, with a workers’ sample that has participated in an online training—confirms the importance of keeping those items in the instrument. Plus, as cognitive and behavioral strategies, they were significant predictors of training effectiveness, appearing to be differential strategies to help learning in virtual settings.

All types of learning strategies were related to training transfer in self-evaluation, specially elaboration and practical application; and the cognitive and help-seeking strategies contributed to explain training transfer in hetero-evaluation. The participants that thought around new material and its implications, looked for connections between course material and their previous knowledge, and identified daily situations where they could try the material out in practice, achieved the best transfer results, considering their overall performance. Such strategies are compatible with the sample of participants who are workers: relating the new knowledge to the one already obtained and trying to realize the usefulness and applicability that it will have in practical daily work activities might be of great importance. Coincidentally, these strategies (elaboration and practical application) were the most frequently used by the sample, scoring the highest means according to the descriptive results, along with self-regulatory strategies of emotion and motivation control.

On the other hand, behavioral strategies (interpersonal and written help-seeking) showed the lowest means, indicating that neither did the participants research other sources of information (web sites, documents, instruction manuals, computer programs, etc.), apart from those available in the course material nor did they look for other co-workers’ help. The absence of interactive tools can explain the low frequency on the proactive behavior of requesting other people’s help and asking them questions about the content of the course material. It may point to the fact that the material might have been enough to resolve any doubts, or to the low complexity of the course, which did not demand further explanations either from other people or from other materials. This finding deserves attention as this kind of strategy contributed to the occurrence of training transfer, affecting the overall job performance.

Regarding the inferior explanation of the self-regulatory strategies on performance results, some reasonable justifications are related to the nature (predominantly cognitive), short duration (2 h), and low complexity of the training. It may not have required the use of strategies that prevent dispersions of concentration caused by anxiety feelings and motivation control, while the learning goals were very simple—although descriptive results inform that these strategies were frequently used by the sample during the course.

Knowing the most effective learning strategies that influence the subsequent process of applying the new skills at work can help training designers or Human Resources Development managers in charge of planning, offering, and evaluating training programs, to guide and encourage trainees to use the most appropriate strategies and identify the (un)successful ones, reconsidering and improving the steps to be taken during the learning process to achieve positive results (see Salas, Tannenbaum, Kraiger, & Smith-Jentsch, 2012). The training design itself should take into account the successful strategies and facilitate, through training planning, exercises, assignments, simulations, etc., their use.

Due the fact that the adoption, use, and influence of learning strategies seem to depend greatly on the characteristics of the sample, course, and organization, some limitations of the study may be the impossibility of generalizing results, or the short duration and complexity of the training evaluated, which surely determined the strategies adopted by the sample and the results obtained. Thus, we suggest that additional studies with the measure should be conducted with other samples and organizations, and the relationships of the learning strategies with other workplace variables should be analyzed.

Future research must continue investigating learning strategies, as they are variables that influence the effectiveness of training and job performance. Beyond that, it is possible to occur a change in the factor structure of learning strategies scales depending on the previously cited aspects, which might also be further explored in forthcoming research.

## Conclusions

In a nutshell, this investigation fills a gap with respect to providing a specific instrument to evaluate e-learning, a context that lacks of validated and reliable measures. From a methodological standpoint, it contributes to the training field, by producing the LSS, with validity evidences (factorial structure) and reliability that can be either used in organizational and academic contexts. Besides, a synthetic version, with no items perceived as semantically identical, is easy and clear to respond. From a theoretical perspective, learning strategies, by providing trainees with skills that help them transfer successfully back to the workplace, are an important cognitive-behavioral and self-regulation variable that might be considered when planning instructional designs. Moreover, our findings show the importance and relevance of all learning strategies, including self-regulatory procedures, to explain the effectiveness of training and training transfer in online courses.

## Abbreviations

BIC: Bayesian Information Criterion
CFA: Confirmatory Factor Analysis
CFI: Comparative Fit Index
CMIN/DF (*χ*^*2*^*/df*): Chi-square/degrees of freedom
GFI: Goodness of Fit Index
ICT: Information and Communication Technologies
LSS: Learning Strategies Scale
RMSEA: Root Mean Square Error Approximation
RMSR: Root Mean Square Residual
SPSS: Statistical Package for the Social Science
TLI: Tucker Luwis Index
